# (*E*)-4-Bromo-*N*-(2,3-dimeth­oxy­benzyl­idene)aniline

**DOI:** 10.1107/S1600536810029880

**Published:** 2010-08-04

**Authors:** Karla Fejfarová, Emanuel Makrlík, Aliakbar Dehno Khalaji

**Affiliations:** a Institute of Physics of the ASCR, v.v.i., Na Slovance 2, 182 21 Praha 8, Czech Republic; b Faculty of Applied Sciences, University of West Bohemia, Husova 11, 30611 Pilsen, Czech Republic; c Department of Chemistry, Faculty of Science, Golestan University, Gorgan, Iran

## Abstract

The title Schiff base compound, C_15_H_14_BrNO_2_, was prepared by the condensation of 2,3-dimeth­oxy­benzaldehyde with 4-bromo­aniline. It adopts an *E* configuration with respect to the C=N bond. The dihedral angle between the two aromatic rings is 56.79 (8)°. Weak C—H⋯O and C—-H⋯π bonds can be found in the crystal structure.

## Related literature

For applications of Schiff-base compounds, see: Yildiz *et al.* (2008 ▶); Hijji *et al.* (2009 ▶); Karakas *et al.* (2008 ▶). For related structures, see: Khalaji *et al.* (2007 ▶, 2009 ▶); Khalaji & Harrison (2008 ▶); Khalaji & Simpson (2009 ▶). For bond-length data, see: Allen *et al.* (1987 ▶).
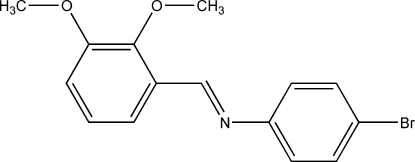


## Experimental

### 

#### Crystal data

C_15_H_14_BrNO_2_
*M* *_r_* = 320.2
Orthorhombic, 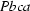

*a* = 13.9978 (2) Å
*b* = 7.0557 (1) Å
*c* = 27.3758 (4) Å
*V* = 2703.75 (7) Å^3^
*Z* = 8
Cu *K*α radiation
μ = 4.13 mm^−1^
*T* = 120 K
0.55 × 0.33 × 0.23 mm

#### Data collection

Oxford Diffraction Xcalibur diffractometer with an Atlas (Gemini ultra Cu) detector
Absorption correction: analytical (*CrysAlis PRO*; Oxford Diffraction, 2009 ▶) *T*_min_ = 0.365, *T*_max_ = 0.698
24799 measured reflections
2365 independent reflections
2178 reflections with *I* > 3σ(*I*)
*R*_int_ = 0.041

#### Refinement

*R*[*F* ^2^ > 2σ(*F* ^2^)] = 0.030
*wR*(*F* ^2^) = 0.123
*S* = 1.82
2365 reflections
172 parameters
H-atom parameters constrained
Δρ_max_ = 0.28 e Å^−3^
Δρ_min_ = −0.31 e Å^−3^

### 

Data collection: *CrysAlis PRO* (Oxford Diffraction, 2009 ▶); cell refinement: *CrysAlis PRO*; data reduction: *CrysAlis PRO*; program(s) used to solve structure: *SIR2002* (Burla *et al.*, 2003 ▶); program(s) used to refine structure: *JANA2006* (Petříček *et al.*, 2006 ▶); molecular graphics: *DIAMOND* (Brandenburg & Putz, 2005 ▶); software used to prepare material for publication: *JANA2006*.

## Supplementary Material

Crystal structure: contains datablocks global, I. DOI: 10.1107/S1600536810029880/bt5308sup1.cif

Structure factors: contains datablocks I. DOI: 10.1107/S1600536810029880/bt5308Isup2.hkl

Additional supplementary materials:  crystallographic information; 3D view; checkCIF report

## Figures and Tables

**Table 1:** Hydrogen-bond geometry (Å, °) *Cg*1 is the centroid of the dimeth­oxy-substituted aromatic ring C2–C7.

*D*—H⋯*A*	*D*—H	H⋯*A*	*D*⋯*A*	*D*—H⋯*A*
C12—H12⋯O2^i^	0.96	2.48	3.425 (3)	167
C7—H7⋯*Cg*1^ii^	0.96	2.84	3.680 (2)	147
C14—H14⋯*Cg*1^iii^	0.96	2.77	3.618 (2)	147

Symmetry codes: (i) 
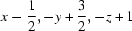
; (ii) 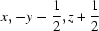; (iii) 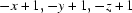.

**Fig. 1.**
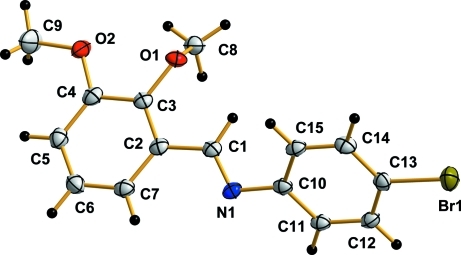
The molecular structure of C15H14BrNO2 with atom-labeling scheme. Displacement ellipsoids are drawn at the 50% probability level.

**Fig. 2.**
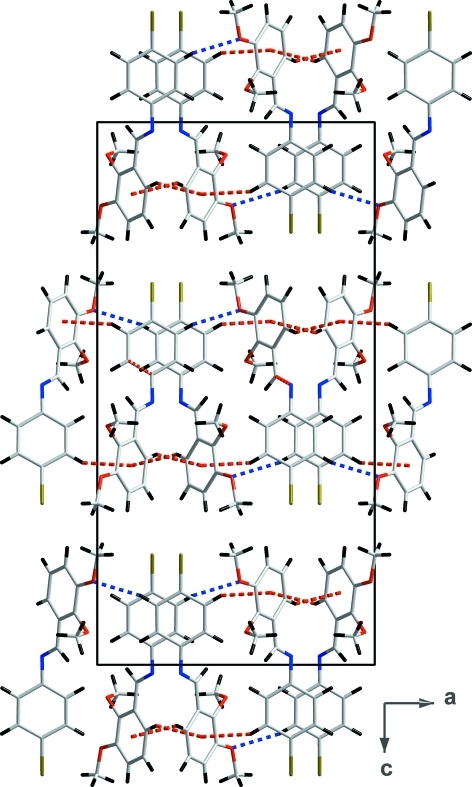
Crystal packing of title coumpound viewed along the b axis. Hydrogen bonds are displayed as blue dashed lines, C—H···π interactions as red dashed lines.

## supplementary crystallographic information

### Comment

The condensation reactions of carbonyl compounds with amines have been extensively used for preparation of the Schiff-base compounds (Yildiz *et al.*, 2008; Hijji *et al.*, 2009; Karakas *et al.*, 2008) which have an importance in diverse fields of chemistry due to their antimicrobial activity (Yildiz *et al.*, 2008), anion sensor properties (Hijji *et al.*, 2009) and applications in nonlinear optic (Karakas *et al.*, 2008). As a continuation of our work on the synthesis and structural characterization of Schiff-base compounds (Khalaji *et al.*, 2007; Khalaji & Harrison, 2008; Khalaji & Simpson, 2009; Khalaji *et al.*, 2009), herein, we report the synthesis and crystal structure of (*E*)-4-bromo-*N*-(2,3-dimethoxybenzylidene)aniline (1).

An *ORTEP* plot, with the atomic numbering scheme is depicted in Fig. 1. Bond lengths in the title compound are in normal range (Allen *et al.*, 1987). The C1—N1 and C10—N1 bond lengths of 1.279 (3), 1.416 (3) Å, respectively, conform to the value for a double and single bonds and they are comparable with the corresponding bond lengths in similar Schiff-base compounds (Khalaji *et al.*, 2007; Khalaji & Harrison, 2008; Khalaji & Simpson, 2009; Khalaji *et al.*, 2009).

The dihedral angle between the two aromatic rings is 56.79 (8)°, while the plane through the central C10—N1—C1—C2 system is inclined at 8.06 (18)° to the dimethoxyphenyl ring and 48.83 (18)° to the bromobenzene ring.

The methoxy group attached at C3 is twisted away from the C2—C7 benzene ring, with corresponding torsion angles C8—O1—C3—C2 113.2 (2)°, while the methoxy group attached at C4 is coplanar with the C2—C7 ring, as shown by the torsion angle C9—O2—C4—C5 of 0.5 (3)°.

In the crystal, molecules are connected by weak C—H···O and C—H···π interactions into layers stacked along *c* (Fig. 2). The layers are further stabilized by aromatic π-π stacking interactions with centroid-centroid distance of 3.8162 (13) Å.

### Experimental

The title compound was prepared in 88% yield from 2,3-dimethoxybenzaldehyde and 4-bromoaniline as reported elsewhere (Khalaji & Harrison, 2008) and recrystallized from chloroform. Anal. Calc. for C_15_H_14_BrNO_2_: C, 56.27; H, 4.41; N, 4.38%. Found: C, 56.45; H, 4.58; N, 4.62%. IR (KBr pellet, cm-1): 2910–2997 (m, C—H aromatic and aliphatic), 2836 (s, –CH=N–); 1605 (s, C=N), 1417–1578 (C=C aromatic).

### Refinement

All hydrogen atoms were discernible in difference Fourier maps and could be refined to reasonable geometry. According to common practice they were nevertheless kept in ideal positions with C–H distance 0.96 Å during the refinement. The methyl H atoms were allowed to rotate freely about the adjacent C—O bonds. The isotropic atomic displacement parameters of hydrogen atoms were set to 1.5×*U*_eq_ (methyl groups) and 1.2×*U*_eq_ of the parent atom.

### Crystal data

**Table:**

C_15_H_14_BrNO_2_	*F*(000) = 1296
*M**_r_* = 320.2	*D*_x_ = 1.573 Mg m^−^^3^
Orthorhombic, *P**b**c**a*	Cu *K*α radiation, λ = 1.54184 Å
Hall symbol: -P 2ac 2ab	Cell parameters from 17724 reflections
*a* = 13.9978 (2) Å	θ = 3.2–66.7°
*b* = 7.0557 (1) Å	µ = 4.13 mm^−^^1^
*c* = 27.3758 (4) Å	*T* = 120 K
*V* = 2703.75 (7) Å^3^	Prism, colourless
*Z* = 8	0.55 × 0.33 × 0.23 mm

### Data collection

**Table:**

Oxford Diffraction Xcalibur diffractometer with an Atlas (Gemini ultra Cu) detector	2365 independent reflections
Radiation source: X-ray tube	2178 reflections with *I* > 3σ(*I*)
mirror	*R*_int_ = 0.041
Detector resolution: 10.3784 pixels mm^-1^	θ_max_ = 66.8°, θ_min_ = 4.5°
Rotation method data acquisition using ω scans	*h* = −16→16
Absorption correction: analytical (*CrysAlis PRO*; Oxford Diffraction, 2009)	*k* = −8→8
*T*_min_ = 0.365, *T*_max_ = 0.698	*l* = −32→32
24799 measured reflections	

### Refinement

**Table:**

Refinement on *F*^2^	56 constraints
*R*[*F*^2^ > 2σ(*F*^2^)] = 0.030	H-atom parameters constrained
*wR*(*F*^2^) = 0.123	Weighting scheme based on measured s.u.'s *w* = 1/[σ^2^(*I*) + 0.0035999999*I*^2^]
*S* = 1.82	(Δ/σ)_max_ = 0.004
2365 reflections	Δρ_max_ = 0.28 e Å^−^^3^
172 parameters	Δρ_min_ = −0.31 e Å^−^^3^
0 restraints	

### Special details

**Table:**

Experimental. CrysAlisPro, Oxford Diffraction (2009), Analytical numeric absorption correction using a multifaceted crystal model.
Refinement. The refinement was carried out against all reflections. The conventional *R*-factor is always based on *F*. The goodness of fit as well as the weighted *R*-factor are based on *F* and *F*^2^ for refinement carried out on *F* and *F*^2^, respectively. The threshold expression is used only for calculating *R*-factors *etc*. and it is not relevant to the choice of reflections for refinement.The program used for refinement, Jana2006, uses the weighting scheme based on the experimental expectations, see _refine_ls_weighting_details, that does not force *S* to be one. Therefore the values of *S* are usually larger than the ones from the *SHELX* program.

### Fractional atomic coordinates and isotropic or equivalent isotropic displacement parameters (Å^2^)

**Table:**

	*x*	*y*	*z*	*U*_iso_*/*U*_eq_	
Br1	0.301516 (18)	0.35856 (4)	0.297810 (9)	0.03286 (15)	
O1	0.46379 (10)	0.9683 (2)	0.56018 (5)	0.0238 (5)	
O2	0.48573 (11)	1.0730 (2)	0.65342 (6)	0.0293 (5)	
N1	0.30118 (11)	0.5268 (3)	0.51592 (7)	0.0204 (6)	
C1	0.35592 (14)	0.6587 (3)	0.53157 (8)	0.0212 (6)	
C2	0.36519 (14)	0.7060 (3)	0.58364 (8)	0.0210 (6)	
C3	0.41859 (14)	0.8666 (3)	0.59624 (8)	0.0208 (6)	
C4	0.42987 (14)	0.9178 (3)	0.64562 (8)	0.0245 (7)	
C5	0.38574 (16)	0.8110 (4)	0.68186 (8)	0.0278 (7)	
C6	0.33284 (18)	0.6502 (3)	0.66869 (9)	0.0285 (7)	
C7	0.32263 (16)	0.5970 (3)	0.62059 (9)	0.0248 (7)	
C8	0.42620 (16)	1.1557 (3)	0.55295 (9)	0.0279 (7)	
C9	0.5018 (3)	1.1323 (5)	0.70222 (9)	0.0479 (11)	
C10	0.30271 (13)	0.4922 (3)	0.46499 (9)	0.0217 (7)	
C11	0.21645 (16)	0.4759 (3)	0.43955 (8)	0.0215 (7)	
C12	0.21516 (16)	0.4411 (3)	0.38997 (9)	0.0239 (7)	
C13	0.30115 (14)	0.4180 (4)	0.36557 (9)	0.0235 (7)	
C14	0.38827 (15)	0.4325 (3)	0.38971 (8)	0.0248 (7)	
C15	0.38811 (15)	0.4684 (3)	0.43928 (8)	0.0228 (7)	
H1	0.392541	0.729923	0.50828	0.0255*	
H5	0.391454	0.846917	0.715555	0.0334*	
H6	0.30309	0.575572	0.693754	0.0342*	
H7	0.286533	0.485819	0.612357	0.0298*	
H8a	0.445019	1.201521	0.521356	0.0419*	
H8b	0.357749	1.152148	0.554914	0.0419*	
H8c	0.450723	1.238776	0.577735	0.0419*	
H9a	0.553271	1.222056	0.702951	0.0718*	
H9b	0.444871	1.190439	0.714812	0.0718*	
H9c	0.518004	1.024448	0.721929	0.0718*	
H11	0.157197	0.489141	0.456874	0.0259*	
H12	0.155673	0.433057	0.37264	0.0287*	
H14	0.447304	0.417836	0.372267	0.0298*	
H15	0.447782	0.477042	0.45641	0.0273*	

### Atomic displacement parameters (Å^2^)

**Table:**

	*U*^11^	*U*^22^	*U*^33^	*U*^12^	*U*^13^	*U*^23^
Br1	0.0370 (3)	0.0382 (3)	0.0233 (3)	0.00213 (10)	0.00027 (8)	−0.00306 (9)
O1	0.0186 (7)	0.0251 (8)	0.0277 (8)	−0.0003 (6)	0.0057 (6)	−0.0002 (6)
O2	0.0230 (8)	0.0386 (10)	0.0263 (8)	−0.0067 (7)	0.0009 (6)	−0.0076 (7)
N1	0.0158 (9)	0.0199 (10)	0.0256 (11)	0.0023 (6)	−0.0014 (6)	−0.0015 (8)
C1	0.0139 (10)	0.0246 (12)	0.0252 (11)	0.0035 (8)	0.0010 (8)	0.0020 (8)
C2	0.0135 (9)	0.0233 (11)	0.0261 (11)	0.0043 (8)	−0.0008 (8)	0.0001 (9)
C3	0.0135 (9)	0.0245 (12)	0.0243 (11)	0.0041 (8)	0.0011 (8)	0.0004 (8)
C4	0.0165 (10)	0.0283 (12)	0.0289 (12)	0.0034 (9)	−0.0012 (9)	−0.0024 (9)
C5	0.0233 (11)	0.0359 (13)	0.0242 (12)	0.0024 (10)	−0.0008 (9)	−0.0027 (10)
C6	0.0251 (11)	0.0347 (14)	0.0259 (13)	0.0005 (9)	0.0025 (10)	0.0057 (9)
C7	0.0187 (9)	0.0256 (12)	0.0302 (13)	0.0007 (9)	−0.0009 (9)	0.0017 (10)
C8	0.0245 (11)	0.0276 (13)	0.0316 (13)	−0.0026 (9)	0.0002 (10)	0.0026 (9)
C9	0.0532 (19)	0.061 (2)	0.0292 (16)	−0.0202 (15)	0.0065 (11)	−0.0175 (11)
C10	0.0199 (11)	0.0188 (12)	0.0265 (13)	−0.0005 (8)	−0.0002 (7)	0.0029 (9)
C11	0.0160 (10)	0.0212 (12)	0.0274 (13)	0.0005 (8)	0.0003 (8)	0.0024 (9)
C12	0.0187 (10)	0.0238 (13)	0.0291 (13)	−0.0022 (9)	−0.0054 (9)	0.0012 (9)
C13	0.0258 (12)	0.0222 (12)	0.0225 (12)	0.0004 (8)	−0.0002 (8)	0.0043 (9)
C14	0.0182 (11)	0.0266 (13)	0.0296 (12)	0.0041 (9)	0.0043 (9)	0.0012 (9)
C15	0.0159 (10)	0.0222 (12)	0.0302 (12)	0.0001 (8)	−0.0028 (8)	0.0011 (9)

### Geometric parameters (Å, °)

**Table:**

Br1—C13	1.902 (2)	C7—H7	0.96
O1—C3	1.375 (3)	C8—H8a	0.96
O1—C8	1.437 (3)	C8—H8b	0.96
O2—C4	1.362 (3)	C8—H8c	0.96
O2—C9	1.418 (3)	C9—H9a	0.96
N1—C1	1.279 (3)	C9—H9b	0.96
N1—C10	1.416 (3)	C9—H9c	0.96
C1—C2	1.470 (3)	C10—C11	1.399 (3)
C1—H1	0.96	C10—C15	1.397 (3)
C2—C3	1.401 (3)	C11—C12	1.380 (3)
C2—C7	1.403 (3)	C11—H11	0.96
C3—C4	1.408 (3)	C12—C13	1.386 (3)
C4—C5	1.391 (3)	C12—H12	0.96
C5—C6	1.402 (3)	C13—C14	1.391 (3)
C5—H5	0.96	C14—C15	1.380 (3)
C6—C7	1.377 (3)	C14—H14	0.96
C6—H6	0.96	C15—H15	0.96
			
C3—O1—C8	114.26 (16)	H8a—C8—H8b	109.4709
C4—O2—C9	118.40 (19)	H8a—C8—H8c	109.4712
C1—N1—C10	116.48 (19)	H8b—C8—H8c	109.4719
N1—C1—C2	122.9 (2)	O2—C9—H9a	109.4702
N1—C1—H1	118.5696	O2—C9—H9b	109.4705
C2—C1—H1	118.5683	O2—C9—H9c	109.4702
C1—C2—C3	118.01 (19)	H9a—C9—H9b	109.4713
C1—C2—C7	122.51 (19)	H9a—C9—H9c	109.472
C3—C2—C7	119.5 (2)	H9b—C9—H9c	109.473
O1—C3—C2	119.40 (19)	N1—C10—C11	119.44 (18)
O1—C3—C4	120.25 (18)	N1—C10—C15	121.99 (18)
C2—C3—C4	120.3 (2)	C11—C10—C15	118.5 (2)
O2—C4—C3	114.91 (19)	C10—C11—C12	121.1 (2)
O2—C4—C5	125.4 (2)	C10—C11—H11	119.4722
C3—C4—C5	119.7 (2)	C12—C11—H11	119.4722
C4—C5—C6	119.3 (2)	C11—C12—C13	118.9 (2)
C4—C5—H5	120.337	C11—C12—H12	120.5379
C6—C5—H5	120.3378	C13—C12—H12	120.5366
C5—C6—C7	121.4 (2)	Br1—C13—C12	119.89 (16)
C5—C6—H6	119.2822	Br1—C13—C14	118.51 (16)
C7—C6—H6	119.2822	C12—C13—C14	121.6 (2)
C2—C7—C6	119.7 (2)	C13—C14—C15	118.6 (2)
C2—C7—H7	120.1302	C13—C14—H14	120.6854
C6—C7—H7	120.13	C15—C14—H14	120.6846
O1—C8—H8a	109.4703	C10—C15—C14	121.2 (2)
O1—C8—H8b	109.4718	C10—C15—H15	119.378
O1—C8—H8c	109.4712	C14—C15—H15	119.3768
			
C8—O1—C3—C2	113.2 (2)	C10—N1—C1—C2	−177.41 (18)
C8—O1—C3—C4	−70.0 (2)	C1—N1—C10—C11	−132.3 (2)
C9—O2—C4—C3	−179.1 (2)	C1—N1—C10—C15	49.5 (3)
C9—O2—C4—C5	0.5 (3)		

### Hydrogen-bond geometry (Å, °)

**Table:**

Cg1 is the centroid of the dimethoxy-substituted aromatic ring.

**Table:**

*D*—H···*A*	*D*—H	H···*A*	*D*···*A*	*D*—H···*A*
C12—H12···O2^i^	0.96	2.48	3.425 (3)	167
C7—H7···Cg1^ii^	0.96	2.84	3.680 (2)	147
C14—H14···Cg1^iii^	0.96	2.77	3.618 (2)	147

Symmetry codes: (i) *x*−1/2, −*y*+3/2, −*z*+1; (ii) *x*, −*y*−1/2, *z*+1/2; (iii) −*x*+1, −*y*+1, −*z*+1.
